# Healthy Garden Plots? Harvesting Stories of Social Connectedness from Community Gardens

**DOI:** 10.3390/ijerph18115747

**Published:** 2021-05-27

**Authors:** Troy D. Glover

**Affiliations:** Department of Recreation & Leisure Studies, University of Waterloo, Waterloo, ON N2L 3G1, Canada; troy.glover@uwaterloo.ca

**Keywords:** emplotment, community narrative, counter-stories, capital and return deficits

## Abstract

Because of their profound effects on health and wellbeing, particularly their sense of social connectedness, community garden stories warrant the close attention of public health professionals. Efforts to tell these stories, if and when told, often smooth over, intentionally ignore or fail to appreciate vital subplots of social experiences that deserve our collective consideration. Put simply, this article advocates for public health to pay greater attention to the subplots—those secondary strands of the main plotline—of community garden stories. To demonstrate, the plot and subplots associated with the Queen Anne Memorial Garden, a community garden located in a diverse urban neighborhood in a Midwestern American city, are examined. The resultant narrative provides a more complex understanding of the social relationships that formed in and around the community garden under examination. Ultimately, the article shows how sublots weave together alternative interpretations of a story based on different constituents’ experiences silenced by main plotlines and encourage audiences to critically reflect upon their own behaviours.

## 1. Introduction

Social connectedness matters significantly to our health and wellbeing. Defined by Haslam and colleagues as “the sense of belonging and subjective psychological bond that people feel in relation to individuals and groups of others” [1] (p. 1), it results in positive physical, mental and personal health, and affects the communities to which we belong in positive ways [2]. Indeed, we tend to be better off if we believe we have interacted, and actually do interact regularly, with others in our lives who care about us [2]. Moreover, having someone upon whom to rely during stressful life events helps us cope more effectively with them [3]. By contrast, if we feel lonely most or all of the time, our loneliness can affect our health negatively and impair our ability to function effectively in society. Amazingly, the detrimental consequences of social isolation liken to well-established risk factors for mortality, including lack of access to healthcare, poor environmental quality, lack of immunization, injury and violence, obesity, poor mental health, risky sexual behavior, sedentary activity, smoking and substance abuse [4]. Given the health benefits of social connectedness and the negative consequences of social isolation, spaces and places that provide the social infrastructure to build, maintain, and sustain healthy social ties, warrant necessary attention from public health authorities.

Community gardens represent “spheres of sociability” [5] (p. 221) and potentially, though not always, healthy social landscapes to advance public health principles. In addition to advancing environmental equity and sustainability, providing access to fresh foods, contributing to life satisfaction and reducing crime, community gardens facilitate important social outcomes, including social cohesion, community building, community resilience, social capital and social interaction [6,7,8,9,10]. Such outcomes derive from experiencing community gardens socially—that is, in the relationalities in which gardeners engage, particularly with others who belong to their community garden networks. The social connections built during participation in the shared act of gardening, in combination with other social, operational and maintenance activities related to the garden, encourage community gardeners to share and access resources within their garden network, which bolsters community capacity [6]. More than simply a physical space in which to garden, community gardens serve as indispensable vehicles for the realization of community [7].

From a constructionist perspective, community gardens emerge and take on identifiable meaning through everyday interactional processes of social definition, signification and production between and among community garden members. The symbolic and imaginary serve as conceptual tools that define the nature of the garden and its practices, meanings, collective identities and, ultimately, social reality, thereby lending meaning to community gardening experiences of self–other relations [11]. “Quite literally,” wrote Bessant, “people think, talk, and act community into existence in the course of their everyday interactions” [11] (p. 471). A *community* garden, therefore, transcends its material form to exist “in its members’ perception of the vitality of its culture,” thereby “making it a resource and repository of meaning, and a referent of their identity” [12] (p. 118). This perception arguably takes the form of stories told about community gardens, stories that “bind individuals together as they recognize they have common experiences that share their identity and their linked futures” [13] (p. 238). Exploring these stories offers a way to understand the culture and context of community gardens and their profound effects on gardeners’ health and wellbeing, particularly their sense of social connectedness. For this reason, community garden stories warrant close attention by public health professionals.

In our efforts to celebrate the central plots of community garden stories, however, we sometimes smooth over, intentionally ignore or fail to appreciate vital subplots of social experiences that are not common knowledge, but still deserve our collective attention. Narratives, by their very nature, are “opportunistic and incomplete” [14] (p. 7). As a result, their telling can privilege certain plots and omit important, yet less known, subplots that reflect power dynamics, thereby impacting the wellbeing of the various characters involved as well as the story’s audience. This article advocates for public health to pay greater attention to the subplots of community garden stories. Public health narratives about community gardens can shift the way people understand “a range of significant relationships,” “inject new and disruptive narratives of change” into given populations, and “promote healthier behaviors” [15] (p. 500), but they can also demoralize, demonize and render invisible accounts that counter and contrast an accepted shared narrative. As proponents of public health, we need to be intentional about harvesting the subplots of community garden stories, along with their plots, with the aim to transform community dynamics and to ensure healthier social dynamics among gardeners. Sometimes unhealthy social dynamics are at play. To demonstrate, I conduct the “narrative-under-analysis” [16] that follows—that is, I treat the narratives that follow as the objects of study—to examine the plots and subplots associated with the Queen Anne Memorial Garden, a community garden located in a diverse urban neighborhood in a Midwestern American city [7,17]. The narratives under scrutiny stem from a qualitative study for which interviews were conducted with fourteen members of a neighbourhood association, split evenly between male and female participants who represented a variety of socio-economic and racial/ethnic backgrounds, involved in the creation of the garden. The plot and subplot presented offer insights into the complexity of community garden stories.

## 2. Narratives as Sources of Empowerment

Stories can be empowering. As Talley wrote, “stories and metaphors are seen as tools for empowerment, valuable social and cultural assets containing knowledge about resources for building community, solving problems, creating resilience, and galvanizing action” [18] (p. 415). To this end, Rappaport sensitized those interested in advancing community empowerment about how dominant cultural narratives, those stories about persons, places or things that contain consistent storylines and thematic content across individuals and settings, affect our personal stories, the autobiographical accounts of our own personal history [19]. Dominant cultural narratives, he explained, transmit through major socializing institutions of culture (e.g., mass media, schools, churches, public health campaigns) and in conversation with others, and affect our general convictions, principles, and identities. Embedded in larger cultural and social frameworks, they reflect hegemonic views, which maintain the status quo. Accordingly, they contain the potential to mislabel, disempower and repress people. Those affected can often internalize and believe these dominant cultural narratives, which show up in their personal stories. Consequently, dominant cultural narratives can have negative implications for collective and self-identity.

What Rappaport called community narratives, however, represent counter-narratives that resist dominant cultural narratives. Community narratives offer alternative stories of the collective experience and knowledge of a specific group of people, which reflects a shared experience that contrasts with dominant cultural narratives and depicts the group in more inspiring ways. Accordingly, community narratives possess counterhegemonic, subversive and liberatory possibilities, for they can be mobilized to change the personal stories of those who feel marginalized by dominant cultural narratives.

The community narrative I offer below provides an aggregation of oral accounts collected about events common to a group of community garden participants as a chronicle of the history of the Queen Anne Memorial Garden. By documenting the narrative content and structure of the community garden, I focus on the plot of the story—that is, the thematic thread of the narrative that gives significance to the progression of events. Using what Ricoeur referred to as narrative emplotment, the process I utilized brings the diverse elements of the Queen Anne Memorial Garden into an imaginative order, in just the same way as does the plot of a story [20]. The significance of the community garden, as a source that drove collective action and brought a neighborhood together, becomes apparent within the context of its plot.

## 3. The Plot of the Queen Anne Memorial Garden

The story of the Queen Anne Memorial Garden began with a neighborhood in serious decline as a remnant of “blockbusting” practices during the mid-to-late 20th century, practices that convinced white homeowners to sell their properties and relocate elsewhere for fear of Black people moving into their neighbourhood. During that period, an influx of low-income residents, the flight of higher income residents and the eventual appearance of drugs, prostitution and gang activity led to poor living conditions for those living in the neighborhood. Neighborhood residents, not surprisingly, felt helpless. To cope with the traumatic change in their surroundings, they resigned themselves to live their lives as if nothing out of the ordinary was happening, even though the circumstances were grim.

Over time, the neighborhood’s reputation within the city diminished tremendously. Outsiders and real estate agents viewed the neighborhood as a dangerous place to live or visit, and lenders began to refrain from approving loans for neighborhood properties. Residents met the negative reputation with growing frustration, but also a grave sense of hopelessness.

After years of resigned acceptance, however, a group of strong-willed and determined residents, made up of mostly white homeowners, assembled to question the normalcy of the activities that surrounded it, eventually working through its fears to address the serious crime in the neighborhood and initiate neighbourhood revitalization. With a commitment to collective action, the group turned its attention toward creating a neighborhood association through which it could concentrate its efforts.

The neighborhood association began by targeting the built environment in the neighborhood. In its first act as a formal body, the association demanded new sidewalks from the city. To the members’ delight, the city agreed to their request. The association’s success empowered the group to ramp up its efforts further. The victory also inspired other residents to join the association, though the group remained relatively small throughout its existence. Nevertheless, the sidewalk victory motivated neighbors to embark upon several other ambitious projects designed to revitalize the neighborhood, including a community policing effort, neighborhood watch, an email discussion group, a neighborhood newsletter, a festival and a community garden project.

With a continued focus on its build environment, the association set its sights on a vacant lot at a busy corner within the center of the neighborhood. The lot attracted several “unsavory characters,” many of whom came from outside of the neighborhood, who loitered on the corner and engaged in a variety of illicit activities. Concerned about the state of the lot, the neighborhood association decided to mount a collective effort to get rid of the individuals who frequented the vacated property. To this end, it convinced the city to lease the lot to the neighborhood association for $1 per year. With the lot in their possession, the association members decided to clean up the site and build a community garden. They named the garden posthumously after Queen Anne, an elderly African American activist who lived in the neighborhood for years and served as one of the first presidents of the neighborhood association.

The Queen Anne Memorial Garden proved to be an attractive way for residents to assist with the association’s efforts to revitalize the neighborhood. While other methods the residents used to address crime in the neighborhood appeared confrontational (e.g., safety patrols), the Queen Anne Memorial Garden offered residents a more calming and mollifying activity in which to get involved, especially for African American residents who feared retribution from those the neighborhood association aimed to displace. The garden made a positive impression within the community, not just inside the neighborhood but also among outsiders who came to see the neighborhood in a more positive light. The garden achieved its primary aim by encouraging the group of unwelcome individuals who frequented the lot to abandon it. Neighbors who lived in close proximity to the garden delighted in the change. Ultimately, the garden became an important symbol in their struggle to reclaim their neighborhood. The story of the Queen Anne Memorial Garden, as a result, served as what one resident described positively as “basically propaganda to show this is an alive neighborhood”.

## 4. Narratives as Sources of Empowerment for Whom?

The story described above fits into a basic plot structure: an inner-city neighborhood experiences severe economic and social decline, it becomes plagued by unwelcome illicit activities that threaten the quality of life of residents, and the residents resign themselves to live with their unfortunate conditions. Eventually, a group of determined residents, fed up with its circumstances, acts collectively to reclaim its neighborhood; the group builds a community garden that displaces the source of many of the unwanted behaviours and the success of the garden strengthens neighborhood social ties and creates greater community capacity that residents can leverage to address crises that emerge in the neighborhood. In this sense, the story of the Queen Anne Memorial Garden fits the common narrative that community gardens can revitalize and reclaim wasted urban areas, which subsequently result in increased community pride, political advocacy and social exchanges [10]. Using Rappaport’s framework, the dominant cultural narrative that depicted the neighborhood as a bad place in which to live infiltrated the personal stories of the residents, but the creation of the Queen Anne Memorial Garden offered residents a community narrative counter to the dominant cultural narrative to provide proof, as it were, that a group of residents could take some measure of control over its collective circumstances by working together.

The community narrative told above worked effectively to mobilize residents. All residents with whom I spoke underscored this narrative structure and expressed their genuine pleasure with what they characterized as the main outcome of the community garden project: a drastically improved social environment within the neighborhood. With this in mind, it would be understandable to leave the narrative as it is. It does its job by assuring residents they live in a good neighborhood. It reveals they do not belong to a group on the margins, and it inspires a shared vision with which to mobilize further action. With these ends in mind, the narrative of the Queen Anne Garden shows the potential of community garden projects for advancing the social aims of a neighborhood.

But what subplots remain untold? What can the secondary strands of the main plot tell us, if anything? More than enabling people to take greater control over their individual health, health promotion aims to address the social determinants of health and wellbeing [21]. Accordingly, delving further into the subplots of a community narrative has the potential to disrupt structural barriers and shift attention toward organizations, systems and environments whose practices can prevent ill health and/or promote better health [22]. Plots can construct reality in ways that maintain the hegemonic order, therein rationalizing oppression and evoking little, if any, self-examination of the oppressor. As a form of intentional resistance to this premise, subplots build a common culture of shared understandings among minority communities whose voices are missing from the main plotline, while simultaneously questioning/rejecting and accepting plotlines, which are challenged through the effective depiction of injustice. Seeking to reveal overlooked threads of storylines, as I hope to show, can expose silences underpinned by disconcerting power dynamics that warrant attention for health promotion purposes.

## 5. Subplots of the Queen Anne Memorial Garden

Leaders involved in the Queen Anne Memorial Garden credited the “tight neighborhood network and people all pulling together to try to improve the situation” as the source of its success. They spoke about how the garden opened up new and different opportunities for neighbors to talk and network, which led to further socializing outside of the community garden project. Moreover, neighborhood association members viewed the social circle that formed to create the community garden as a resource upon which they drew when faced with other issues in their neighborhood. Evidence showed the community garden project led to greater stockpiles of social capital within the neighborhood.

The diversity of the group dedicated to building and maintaining the Queen Anne Memorial Garden stood out as a genuine marker of success. The inner-city neighborhood where the garden was located, the oldest neighborhood in the city, boasted a variety of housing options and a rich mix of neighbours representing different racial, ethnic, and socioeconomic groups. While residents largely hunkered down and kept to themselves prior to the creation of the neighborhood association, the community garden encouraged social mixing among a diverse group of residents who wanted to make a change to their neighborhood in a more conciliatory manner. In particular, the prevalence of participation among Black residents in the garden was, for neighborhood association leaders, a positive sign the neighborhood was improving.

While many residents joined the community garden effort to make their neighborhood a safer place, those not in leadership positions associated with the neighborhood association confided they felt somewhat cynical about the motives of the leaders involved. More specifically, the feeling that neighborhood association leaders, a group of white homeowners in the neighborhood, wanted to increase their property values pervaded among the group of largely Black and Latino renters who volunteered to assist with the community garden project. The neighborhood association’s aggressive assault on crime, furthermore, worried nonwhite neighbors the association would eventually turn its attention on other residents who failed to fit the picture of an ideal neighbor. This concern dissuaded some residents from participating in neighborhood association activities altogether. For them, the neighbors leading the charge belonged to an exclusive group of residents committed only to their own ends. As a result, they continued to view the garden as “the white folks’ project.”

Though neighborhood association leaders acknowledged race as a divide they sought to overcome, some still criticized African American residents for not taking a more active role in the neighborhood association’s efforts. What they viewed as harmless decisions about the community garden worked to create further social divisions. For example, the introduction of a lock to the garden gate to address acts of vandalism against the garden by those displaced by the garden effort was put into place with seemingly benign intentions, but served only to further complicate relations between the white homeowners in leadership positions (who controlled the key to the lock) and other neighborhood residents, particularly African American renters, who wanted access. Effectively, the locked gate denied admittance to what was meant to be common neighborhood space. Moreover, by controlling the key to the lock, neighborhood association leaders, perhaps unwittingly, claimed possession over the garden and excluded others from enjoying a sense of ownership.

The absence of vegetables in the Queen Anne Memorial Garden presented yet another source of contention. By choosing to make the garden exclusively ornamental, the neighborhood association leaders opted for the garden to fulfill its purpose as a beautification project. Though seemingly a trivial and inconsequential decision to the white homeowners, to the Black renters with no backyards of their own, gaining access to a public space to grow vegetables and fruits would have made an enormous difference to their quality of life. Unfortunately, the decision to make a flower garden proceeded without any meaningful consultation with those outside of the leadership group. As a result, the community garden project, once again, was perceived as something exclusive and imposed on those without a voice. The consequences of excluding others in the planning process meant less support for the garden than the neighborhood association had desired. Ultimately, those not in leadership positions praised the construction of the garden, but felt not fully connected to it because of the lack of consultation.

## 6. Discussion

While the positive benefits associated with the Queen Anne Memorial Garden seemed to make it worth the social investment for all who contributed to it, Black members of the community garden group, in particular, clearly accessed resources embedded in the network unequally. The sources of this unequal access to resources seemingly stemmed from what Lin referred to as capital and return deficits [23]. First, the African American residents involved with the community garden appeared to enter into the garden network with an unequal footing (i.e., capital deficit), relative to their white counterparts, because of their racial status in American society and their racial ties to the undesirable group of individuals the neighborhood sought to displace. Second, Black renters reaped different rewards for their contributions to the garden project, despite making the same relative investments of time and effort as other groups (i.e., return deficit). Ultimately, Black members of the garden group accessed only partial entry, if any, into the core social circle that controlled decision-making over the garden. Whether the leaders of the neighborhood association (i.e., white homeowners) limited access to resources within the network intentionally or unintentionally remains unclear, but the racialized members of the garden group were undeniably left out. The key to the garden gate represented a powerful metaphor for exclusion as nonwhite renters found themselves literally and figuratively locked out of the garden.

By examining the garden story beyond its main plot and using what Rosiek and Snyder referred to as a “stories-as-evidence-of-things-overlooked” approach [14], the subplots of the garden story exposed clear power imbalances in the garden network. Moreover, it introduced conflicting voices in the narrative’s (re)production, allowed for the sharing of alternative truth-claims, and exposed political struggle embedded within the Queen Anne Memorial Garden narrative. According to hooks, privileging those who “see” the world differently is the easiest and most direct way to consciously deconstruct hegemonic ways of knowing [24]. Exposing subplots enabled this counter-hegemonic process to unfold.

The addition of subplots, moreover, revealed the complex social realities of the Queen Anne Memorial Garden. The garden narrative, with its plot and subplots, shows a community garden can produce not just affiliation, but also distance. While positive feelings emerged among those involved with Queen Anne Memorial Garden, so too did social distinctions and power imbalances. Social divides were bridged, yet at the same time remained intact and were perhaps even solidified further. These outcomes made it hard to discern the line between experiencing *comfort* and feeling *comfortable* among the leaders of the community garden group. Whereas the former suggests security, the latter implies avoidance of tension and discomfort, which is often achieved by encouraging those involved to concentrate on the positive narrative shared. But positive stories are not unlike colorblind narratives insofar as they ignore race and ethnicity to cause inconsistencies in the aims and ambitions of collective actions. They impose white values and white conduct on nonwhite participants to preserve the status quo.

In sharing subplots to reveal the social complexities of community gardens, stories enable, foster, and/or provoke audiences to consider new subject positions. They intervene in the audience experience and potentially transform the audience’s response to institutionalized racism, a social determinant of health. “If our goal is to change behavior,” wrote Petraglia in reference to the relevance of stories in public health, “we have to know whether we are either aligning our stories with existing social narratives or purposely seeking to inject new and perhaps disruptive narratives of change into a given population” [15] (p. 497–498). As regards the latter, a narrative that includes plots and subplots provides the conceptual, intellectual and evidential bases for critical reflection. Critical reflection, here, means something more than inviting introspection; it means challenging the status quo. By interrogating privileges, exposing misuses of power and challenging decision-making and (in)action, stories identify problems that warrant attention and open up new ways of seeing things. They envision ways in which life should be lived and identify the roles, practices or policies that potentially move communities closer to them, not necessarily to please audiences, but to challenge them to think and act differently.

## 7. Conclusions

Community gardens contain the potential to contribute significantly to public health efforts to encourage and support social connectedness. Evidence in the literature reveals they promote social inclusion, social interaction, cohesion, support networks and social capital [6,7,8,9,10]. The main plot of the story of the Queen Anne Memorial Garden generally confirms these outcomes. A closer look at the subplots of its story, however, reveals greater complexity.

Examining subplots of community garden stories, as demonstrated above, can provide more complex understandings of the social relationships that form in and around community garden contexts. While white homeowners established well-developed social connectedness in their work to create the Queen Anne Memorial Garden, their connectedness was itself bifurcated by race. In this sense, subplots offer critical counter perspectives on what otherwise may be perceived as a positive plot-driven narrative. They weave together alternative interpretations of a story based on different constituents’ experiences silenced by main plotlines to illuminate audiences and “open up possibilities for transformative shifts in perception and practice [25] (p. 250). Accordingly, giving greater attention to subplots may encourage people to rethink their behaviours in their own situations. From a public health perspective, then, these counter stories have the potential to “induce a significant shift in one’s worldview and the way the person understands a range of significant relationships and reemplots past experience and future expectations” [15] (p. 500). Consequently, they have the power to draw us in and change our attitudes and opinions” [26] (p. 216).

Determining what storylines constitute a plot versus a subplot warrants acknowledgement and recognition of an author’s subjectivity and narrative privilege, though [27]. That I considered one narrative thread about the Queen Anne Memorial Garden to be the garden story’s main plotline, and others the garden story’s subplots, speaks to my positionality as a white, upper middle-class male researcher who collected, interpreted and (re)presented its various accounts. Moreover, even in adding subplots to this article, other accounts of lived experience continue to remain silenced, namely those of the people displaced by the community garden effort (i.e., the group of so-called “unsavory” individuals who were largely poor, young, and African American). Undoubtedly, these authorial decisions—to view the successful collective action of a group of residents as the primary thematic thread and the challenges experienced by nonwhite actors as secondary or not even important enough to chronicle—reflect my own implicit biases. Not unlike gardeners who select among their harvests the produce they wish to eat, public health researchers discern among the narrative fragments they collect and piece together the story they wish to tell. In so doing, they reflect and shape ideas about health, what affects health, and what could be done to improve health [18]. Do community gardens tell a healthy story about social connectedness? It depends on the story we choose to tell.

## Data Availability

Data are not publicly archived for privacy reasons.
